# Pre-enlistment Anger Attacks and Postenlistment Mental Disorders and Suicidality Among US Army Soldiers

**DOI:** 10.1001/jamanetworkopen.2021.26626

**Published:** 2021-09-27

**Authors:** Diana M. Smith, Alejandro Meruelo, Laura Campbell-Sills, Xiaoying Sun, Ronald C. Kessler, Robert J. Ursano, Sonia Jain, Murray B. Stein

**Affiliations:** 1 Department of Psychiatry, University of California, San Diego, La Jolla; 2 Biostatistics Research Center, Herbert Wertheim School of Public Health and Human Longevity Science, University of California, San Diego, La Jolla; 3 Department of Health Care Policy, Harvard Medical School, Boston, Massachusetts; 4 Center for the Study of Traumatic Stress, Department of Psychiatry, Uniformed Services University of the Health Sciences, Bethesda, Maryland; 5 Psychiatry Service, Veterans Affairs San Diego Healthcare System, San Diego, California; 6 Herbert Wertheim School of Public Health and Human Longevity Science, University of California, San Diego, La Jolla

## Abstract

**Importance:**

Anger is linked to adverse outcomes in military populations; however, whether pre-enlistment anger attacks are associated with postenlistment mental disorders and suicidality is unknown.

**Objective:**

To explore the associations of pre-enlistment anger attacks with postenlistment mental health.

**Design, Setting, and Participants:**

In this observational cohort study, the Army Study to Assess Risk and Resilience in Servicemembers (Army STARRS) New Soldier Study (NSS) surveyed soldiers entering basic training from April 2011 to November 2012, with a subsample recruited for wave 1 of the STARRS Longitudinal Study (STARRS-LS) (conducted September 2016 to April 2018). Participants were recruited from 3 US Army installations for the NSS survey. Those who were subsequently contacted for STARRS-LS completed the follow-up survey via web or telephone. Prospective analyses were based on a weighted NSS subsample included in wave 1 of STARRS-LS. Data were analyzed from May 22, 2020, to March 17, 2021.

**Exposures:**

History of anger attacks at baseline (NSS). Survey responses were used to classify new soldiers as having nonimpairing anger attacks (>2 attacks without interference in work or personal life), impairing anger attacks (>2 attacks with interference in work or personal life), or no significant history of anger attacks.

**Main Outcomes and Measures:**

Baseline analyses examined sociodemographic and clinical correlates of a history of anger attacks. Prospective logistic regression models estimated associations of baseline history of anger attacks with new onset and persistence of posttraumatic stress disorder, major depressive disorder (MDD), generalized anxiety disorder (GAD), panic disorder, mania/hypomania, substance use disorder, suicidal ideation, and suicide attempt at wave 1 of STARRS-LS.

**Results:**

Of the 38 507 baseline participants (83.0% male and 17.0% female; mean [SD] age, 20.97 [3.57] years), 6216 were selected for and completed wave 1 of the STARRS-LS. Baseline prevalence (SE) of nonimpairing and impairing anger attacks was 8.83% (0.16%) and 5.75% (0.15%), respectively. Prospective models showed that impairing anger attacks were associated with new onset of MDD (adjusted odds ratio [AOR], 1.98; 95% CI, 1.31-2.99), GAD (AOR, 2.39; 95% CI, 1.66-3.45), panic disorder (AOR, 2.02; 95% CI, 1.34-3.05), and suicidal ideation (AOR, 2.11; 95% CI, 1.45-3.07). When baseline psychiatric comorbidity was controlled for, impairing attacks remained associated with onset of GAD (AOR, 1.75; 95% CI, 1.19-2.58) and suicidal ideation (AOR, 1.62; 95% CI, 1.09-2.42). Anger attacks were not significantly associated with persistence of pre-enlistment mental disorders.

**Conclusions and Relevance:**

The findings of this study suggest that a pre-enlistment history of impairing anger attacks may be associated with elevated risk of developing GAD, MDD, and suicidality after enlistment. Detection of impairing anger attacks could aid in assessing psychiatric risk in new soldiers.

## Key Points

**Question:**

Is a history of anger attacks before enlistment associated with new onset and persistence of mental disorders and suicidality after enlistment among new US Army soldiers?

**Findings:**

In a cohort study of 38 507 new soldiers, a pre-enlistment history of impairing anger attacks (ie, attacks causing life interference) was significantly associated with postenlistment onset of major depression, generalized anxiety disorder (GAD), panic disorder, and suicidal ideation. These associations were partly explained by psychiatric comorbidity; however, impairing anger attacks were independently associated with new onset of GAD and suicidal ideation.

**Meaning:**

These findings suggest that detection of impairing anger attacks could aid in assessing elevated risk of developing anxiety disorders, depression, and suicidality after enlistment.

## Teaser

This cohort study uses data from the Army STARRS New Soldier Study to investigate whether pre-enlistment anger attacks are associated with new onset and persistence of mental disorders and suicidality after enlistment in a US Army sample.

## Introduction

Anger is experienced by all humans to varying degrees, including in situations where it may be adaptive. However, excessive anger can be pathological, and extreme cases can indicate psychiatric illness. Intermittent explosive disorder (IED), characterized by repeated outbursts of verbal or physical aggression (anger attacks), affects approximately 4% to 7% of the US population in their lifetimes.^1,2^ In military populations, it is even more common, with 11.4% of active duty Army personnel meeting *Diagnostic and Statistical Manual of Mental Disorders, Fourth Edition* (*DSM-IV*) criteria for past 30-day IED.^3^ Notably, several studies^4,5,6^ have found associations between IED and subsequent suicidal ideation and attempts in soldiers.

The New Soldier Study (NSS) of the Army Study to Assess Risk and Resilience in Servicemembers (Army STARRS)^7^ also found high prevalence (14.6%) of lifetime *DSM-IV* IED among soldiers starting basic training.^8^ Since the development of the NSS survey, the criteria for IED have been revised for the *Diagnostic and Statistical Manual of Mental Disorders, 5th edition* (*DSM-5*).^9^ Anger attacks remain the cardinal feature of IED, but the *DSM-5* definition requires greater frequency of attacks and significant life interference or marked distress.^10^ Despite these definitional changes, the study of anger attacks stemming from *DSM-IV* criteria remains relevant because evidence suggests that a range of anger-related problems (eg, aggression, hostility) are associated with adverse outcomes among service members. For example, anger problems evaluated outside *DSM-IV* or *DSM-5* IED criteria have been associated with suicidality^11^ and have been found to mediate the association between posttraumatic stress disorder (PTSD) and suicidality among veterans.^11,12^

Whereas prior studies provide vital information about the risk of suicide-related outcomes in service members with IED and other anger problems,^4,5,6^ further investigation is needed to understand the association between anger and other mental health problems in military populations. Moreover, previous studies have primarily used cross-sectional data; thus, the evidence that anger problems predispose service members to later mental health problems is limited. Accordingly, the present study aimed to examine baseline correlates and psychiatric sequelae of anger attacks in the NSS sample. In particular, we investigated the prospective associations of pre-enlistment anger attacks with new onset and persistence of mental disorders and suicidality after enlistment.

## Methods

### Overview/Participants

The NSS surveyed soldiers beginning basic combat training at 3 US Army installations from April 2011 to November 2012.^7,13,14^ Data collection methods are detailed elsewhere^8^; all Army STARRS survey instruments are available online (https://www.starrs-ls.org/#/page/instruments). After providing written informed consent, participants completed a computer-based self-administered questionnaire that included demographic and diagnostic measures. Recruitment, informed consent, and data collection procedures were approved by the Human Subjects Committees of the Uniformed Services University of the Health Sciences, Bethesda, Maryland, for the Henry M. Jackson Foundation for the Advancement of Military Medicine (the primary grantee), the Institute for Social Research at the University of Michigan, Ann Arbor (the organization collecting the data), Harvard Medical School, Boston, Massachusetts, and University of California, San Diego, La Jolla. This report follows the Strengthening the Reporting of Observational Studies in Epidemiology (STROBE) reporting guideline for cohort studies.

Nearly all (99.9%) selected soldiers consented to the NSS self-administered questionnaire, and 93.5% completed it. Most completers (77.1%) consented to linkage of questionnaire data to their Army/US Department of Defense records; these 38 507 soldiers constituted the eligible sample for the present study. Weights were used to account for potential response bias related to survey completion vs noncompletion and consenting to vs refusing administrative record linkage.^15^

The STARRS Longitudinal Study (STARRS-LS) is a follow-up study of soldiers who participated in Army STARRS surveys; wave 1 data were collected from September 2016 to April 2018. The prospective analysis was conducted in a subsample of 6216 soldiers whose NSS data were successfully linked to STARRS-LS wave 1 data. Analyses of STARRS-LS data incorporated weights that adjusted for the stratified sampling strategy (eMethods in the Supplement). The mean interval from baseline (NSS) to follow-up (STARRS-LS wave 1) was 5.25 (range, 3.85-6.83) years.

### Measures

#### History of Anger Attacks

The NSS survey evaluated anger attacks, described as episodes “when all of a sudden you lost control and either broke or smashed something worth more than a few dollars, hit or tried to hurt someone, or threatened someone.” Episodes were counted as anger attacks only if respondents reported (1) difficulty controlling the aggressive impulse, experiencing attacks in situations where most people would not get angry, or high frequency of attacks (>10 lifetime attacks) and (2) that attacks had occurred when they were not using alcohol or drugs. A history of anger attacks was considered present only if attacks were recurrent; this was determined based on responses to an item assessing how many attacks respondents had experienced in their lifetimes (1 or 2, 3-5, 6-10…to ≥101). Given that the *DSM-5* revision of the IED category included the addition of a life interference/distress criterion, we compared the characteristics and risk profiles of soldiers with a pre-enlistment history of nonimpairing anger attacks vs impairing anger attacks to examine possible clinical implications of this distinction. Soldiers were classified as having nonimpairing anger attacks if they reported more than 2 lifetime anger attacks that had interfered with their work or personal lives a little of the time or none of the time. Soldiers were classified as having impairing anger attacks if they reported more than 2 anger attacks that had interfered some of the time, most of the time, or all/almost all the time. The remainder of the sample was classified as having no significant history of anger attacks.

#### Sociodemographic and Service Characteristics

Age, sex, race and ethnicity, educational attainment, marital status, service component (Regular Army, Guard, or Reserve), and enrollment site were included as covariates in all regression models. In addition, the prospective models adjusted for self-reported deployment status (ever vs never deployed) and military status at follow-up (active duty, activated Guard/Reserve, Guard/Reserve [not currently activated], retired, or separated).

#### Mental Disorders and Suicidality

Respondents to the NSS completed items adapted from the Composite International Diagnostic Interview screening scales^16^ and PTSD Checklist–Civilian Version^17^ to assess lifetime *DSM-IV* diagnoses including PTSD, major depressive disorder (MDD), generalized anxiety disorder (GAD), panic disorder (PD), conduct disorder, oppositional defiant disorder, mania/hypomania, and substance use disorder (SUD). Diagnoses based on the Composite International Diagnostic Interview screening scales and PTSD Checklist–Civilian Version showed satisfactory concordance with diagnoses from structured clinical interviews.^18^ Lifetime suicidal ideation and attempt were assessed using a self-reported version of the Columbia–Suicide Severity Rating Scale.^19^ The cross-sectional analysis examined associations of anger attacks with each mental disorder diagnosis, suicidal ideation, and suicide attempt. For the prospective analysis, we derived 2 composite diagnostic variables, which reflect baseline psychiatric comorbidity. These were lifetime pre-enlistment history of internalizing disorder (yes if lifetime PTSD, MDD, mania/hypomania, GAD, or PD) and lifetime pre-enlistment history of externalizing disorder (yes if conduct disorder, oppositional defiant disorder, or SUD). Disorder groupings were based on evidence from large-scale epidemiological studies.^20,21^

The STARRS-LS wave 1 survey contained analogous measures of mental disorders and suicidality, except that survey sections were updated to reflect *DSM-5* criteria. The outcomes of interest at follow-up were past 12-month PTSD, MDD, GAD, PD, mania/hypomania, suicidal ideation, suicide attempt, and past 30-day SUD (past 12-month SUD was not assessed). In defining outcomes for the prospective analysis, we differentiated between new onset and persistence of conditions. New onset was defined as reporting no lifetime history of a disorder in the NSS survey but meeting criteria for that disorder in the STARRS-LS wave 1 survey. Persistence of a disorder was defined as reporting lifetime history of a disorder in the NSS survey and reporting recent symptoms of that disorder in the STARRS-LS wave 1 survey. We aimed to model new onset and persistence of each outcome but were unable to fit persistence models of PD, mania/hypomania, and suicide attempt owing to low baseline prevalence.

### Statistical Analysis

#### Baseline Analyses

Sociodemographic characteristics of new soldiers with impairing vs nonimpairing vs no anger attacks were compared using Kruskal-Wallis tests for continuous variables and Fisher exact tests for categorical variables. When omnibus test findings were significant, pairwise comparisons were performed. A series of weight-adjusted logistic regression models were fit to estimate the associations of nonimpairing and impairing anger attacks with lifetime mental disorders, suicidal ideation, and suicide attempt (as reported in the NSS survey). These cross-sectional models included covariates for age, sex, race and ethnicity, educational attainment, marital status, and site of basic combat training.

#### Prospective Analyses

Two sets of weight-adjusted logistic regression models were fit to examine the association of pre-enlistment history of anger attacks (measured in the NSS) with new onset and persistence of mental health problems at wave 1 of the STARRS-LS. The first set of prospective models estimated the associations of pre-enlistment anger attacks with postenlistment mental health outcomes after adjustment for sociodemographic and service variables (age, sex, race and ethnicity, educational attainment, marital status, site of basic combat training, deployment history at follow-up, and military status at follow-up). To assess whether any associations between pre-enlistment anger attacks and postenlistment mental health outcomes were explained by psychiatric comorbidity, we fit a second set of prospective models that added controls for lifetime internalizing disorder and lifetime externalizing disorder at baseline. The fully adjusted model of new-onset suicide attempt also controlled for lifetime suicidal ideation at baseline.

Data were analyzed from May 22, 2020, to March 17, 2021. Two-tailed *P* < .05 was considered statistically significant for all analyses except for pairwise comparisons, to which a Bonferroni correction was applied and for which 2-tailed *P* < .017 was considered statistically significant. Analyses were conducted in R, version 3.6.1.^22^

## Results

**Figure 1.**
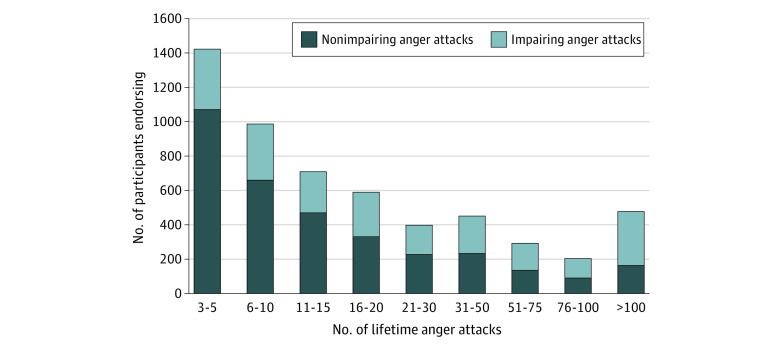
Frequency Distribution Showing Number of Lifetime Anger Attacks Stratified by Impairment Status Among New Soldiers Reporting Recurrent Anger Attacks Anger attack frequency was significantly associated with impairment status, with participants with a history of impairing anger attacks reporting a higher total number of lifetime anger attacks (*P* < .001).

The NSS sample consisted of 38 507 participants (83.0% male and 17.0% female; mean [SD] age, 20.97 [3.57] years). Of these, 6216 participants were selected for and completed wave 1 of STARRS-LS. Weighted prevalence of nonimpairing and impairing anger attacks in the NSS baseline sample was 8.83% (SE, 0.16%) and 5.75% (SE, 0.15%), respectively. Figure 1 shows the distribution of anger attack frequency among soldiers who reported recurrent anger attacks and highlights that soldiers with more frequent attacks were more likely to report impairment (eg, 27.0% of those with impairing attacks reported >50 attacks in their lifetime, compared with 11.6% of those with nonimpairing attacks). Table 1 shows sociodemographic and service characteristics by history of anger attacks. Compared with the group without anger attacks, both anger attack groups were younger, had relatively more male and non-Hispanic White soldiers, and had fewer college-educated and married soldiers. Sociodemographic characteristics of new soldiers with nonimpairing vs impairing anger attacks were similar.

**Table 1.** Distribution and Associations of Baseline Characteristics With Pre-enlistment Anger Attacks

Characteristic	History of anger attacks (N = 38 507)^a^			*P* value^b^	Pairwise comparisons		
	None (n = 32 977)	Nonimpairing (n = 3392)	Impairing (n = 2138)		None vs nonimpairing	None vs impairing	Nonimpairing vs impairing
Age, mean (SD), y	21.07 (3.63)	20.47 (3.18)	20.16 (2.90)	<.001	<.001	<.001	<.001
Sex							
Male	27 165 (82.4)	2963 (87.4)	1838 (86.0)	<.001	<.001	<.001	.14
Female	5812 (17.6)	429 (12.6)	300 (14.0)				
Race and ethnicity							
Hispanic	5121 (15.5)	410 (12.1)	248 (11.6)	<.001	<.001	<.001	.20
Non-Hispanic							
Black	5831 (17.7)	457 (13.5)	275 (12.9)				
White	19 670 (59.6)	2275 (67.1)	1484 (69.4)				
Non-Hispanic other^c^	2355 (7.1)	250 (7.4)	131 (6.1)				
Educational attainment							
GED	3756 (11.4)	399 (11.8)	234 (10.9)	<.001	<.001	<.001	.22
High school	26 923 (81.6)	2841 (83.8)	1825 (85.4)				
College	2298 (7.0)	152 (4.5)	79 (3.7)				
Marital status							
Married	4083 (12.4)	335 (9.9)	210 (9.8)	<.001	<.001	<.001	.96
Unmarried	28 894 (87.6)	3057 (90.1)	1928 (90.2)				
Service component							
Regular Army	18 711 (56.7)	1937 (57.1)	1192 (55.8)	.006	.34	<.001	.12
National Guard	9304 (28.2)	977 (28.8)	669 (31.3)				
Reserve	4962 (15.0)	478 (14.1)	277 (13.0)				

Abbreviation: GED, General Educational Development.

^a^ Unless otherwise indicated, data are expressed as number (%) of participants. Percentages have been rounded and may not total 100.

^b^ Statistical significance of between-groups differences was evaluated using Kruskal-Wallis test for age and Fisher exact test for the rest of the variables.

^c^ Includes American Indian or Alaskan Native, Asian, Native Hawaiian or other Pacific Islander, and other.

**Figure 2.**
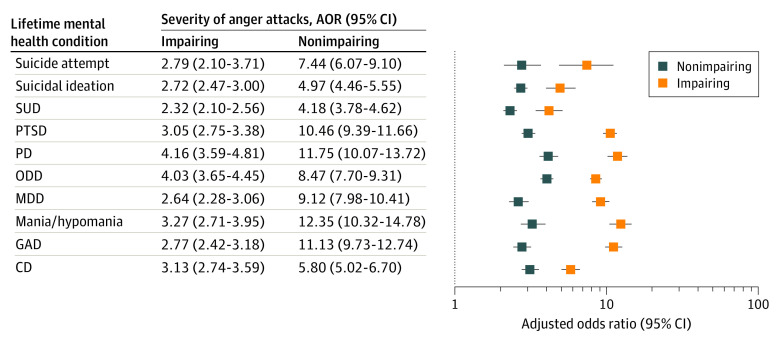
Cross-sectional Associations of a History of Anger Attacks With Other Lifetime Mental Disorders Each point represents the coefficient from a single weight-adjusted logistic model estimating the association of pre-enlistment anger attacks (impairing or nonimpairing) with other pre-enlistment mental health problems. Estimates indicate adjusted odds ratios (AORs). Error bars indicate 95% CIs. All models are adjusted for sociodemographic and Army service characteristics, and the reference group in all cases was soldiers with no lifetime history of anger attacks. Both nonimpairing and impairing anger attacks were significantly associated with all mental health outcomes (all *P* < .001). CD indicates conduct disorder; GAD, generalized anxiety disorder; MDD, major depressive disorder; ODD, oppositional defiant disorder; PD, panic disorder; PTSD, posttraumatic stress disorder; and SUD, substance use disorder.

Figure 2 shows results of logistic regression models assessing the cross-sectional associations of a history of anger attacks with lifetime mental health conditions in the NSS. New soldiers with nonimpairing anger attacks had higher odds of lifetime suicide attempts (adjusted odds ratio [AOR], 2.79; 95% CI, 2.10-3.71), suicidal ideation (AOR, 2.72; 95% CI, 2.47-3.00), SUD (AOR, 2.32; 95% CI, 2.10-2.56), PTSD (AOR, 3.05; 95% CI, 2.75-3.38), PD (AOR, 4.16; 95% CI, 3.59-4.81), oppositional defiant disorder (AOR, 4.03; 95% CI, 3.65-4.45), MDD (AOR, 2.64; 95% CI, 2.28-3.06), mania/hypomania (AOR, 3.27; 95% CI, 2.71-3.95), GAD (AOR, 2.77; 95% CI, 2.42-3.18), and conduct disorder (AOR, 3.13; 95% CI, 2.74-3.59) compared with the group without anger attacks. New soldiers with impairing anger attacks also had higher odds of lifetime suicide attempts (AOR, 7.44; 95% CI, 6.07-9.10), suicidal ideation (AOR, 4.97; 95% CI, 4.46-5.55), SUD (AOR, 4.18; 95% CI, 3.78-4.62), PTSD (AOR, 10.46; 95% CI, 9.39-11.66), PD (AOR, 11.75; 95% CI, 10.07-13.72), oppositional defiant disorder (AOR, 8.47; 95% CI, 7.70-9.31), MDD (AOR, 9.12; 95% CI, 7.98-10.41), mania/hypomania (AOR, 12.35; 95% CI, 10.32-14.78), GAD (AOR, 11.13; 95% CI, 9.73-12.74), and conduct disorder (AOR, 5.80; 95% CI, 5.02-6.70) compared with the group without anger attacks.

The first set of prospective models (partial models in Table 2), which adjusted for sociodemographic and service variables, showed that, in most cases, adjusted odds of the outcomes were significantly elevated for soldiers with impairing anger attacks at baseline, but not for those with nonimpairing attacks. Pre-enlistment impairing anger attacks were associated with new onset of MDD (AOR, 1.98; 95% CI, 1.31-2.99), GAD (AOR, 2.39; 95% CI, 1.66-3.45), PD (AOR, 2.02; 95% CI, 1.34-3.05), and suicidal ideation (AOR, 2.11; 95% CI, 1.45-3.07) (*P* < .001 for all), but not PTSD (AOR, 1.52; 95% CI, 0.90-2.56), mania/hypomania (AOR, 1.26; 95% CI, 0.53-3.00), SUD (AOR, 1.42; 95% CI, 0.70-2.88), or suicide attempt (AOR, 2.77; 95% CI, 0.80-9.60) (*P* > .10 for all). Nonimpairing anger attacks were significantly associated with first suicide attempt (AOR, 2.21; 95% CI, 1.05-4.65; *P* = .04), but not with the other psychiatric outcomes tested. Models investigating suicide attempts at follow-up were limited by the small number of participants reporting suicide attempts. A history of anger attacks at baseline was not associated with persistence of pre-enlistment mental health problems.

**Table 2.** Associations Between Anger Attack and Mental Disorder Variables at Baseline and New Onset of Mental Health Outcomes at Follow-up^a^

Outcome	No. of participants in model	Independent variable of interest, AOR (95% CI)					
		Partial models, anger attacks		Full models			
		Nonimpairing	Impairing	Anger attacks		Internalizing disorder^b^	Externalizing disorder^c^
				Nonimpairing	Impairing		
PTSD	5233	1.07 (0.71-1.62)	1.52 (0.90-2.56)	0.99 (0.64-1.53)	1.24 (0.73-2.10)	1.38 (1.00-1.89)^d^	1.21 (0.90-1.62)
MDD	5629	1.05 (0.78-1.41)	1.98 (1.31-2.99)^e^	0.93 (0.68-1.28)	1.50 (0.97-2.33)	1.55 (1.23-1.96)^f^	1.23 (0.95-1.58)
GAD	5617	0.95 (0.70-1.30)	2.39 (1.66-3.45)^f^	0.83 (0.59-1.17)	1.75 (1.19-2.58)^e^	1.66 (1.30-2.11)^f^	1.22 (0.95-1.55)
PD	6011	1.39 (0.97-2.01)	2.02 (1.34-3.05)^e^	1.17 (0.81-1.70)	1.38 (0.95-2.02)	1.68 (1.33-2.12)^f^	1.32 (0.99-1.76)
Mania/hypomania	5932	1.46 (0.64-3.35)	1.26 (0.53-3.00)	1.30 (0.54-3.14)	0.99 (0.37-2.67)	1.29 (0.77-2.17)	1.31 (0.81-2.12)
SUD	5243	1.52 (0.97-2.37)	1.42 (0.70-2.88)	1.34 (0.85-2.13)	1.04 (0.53-2.05)	1.46 (1.07-1.99)^d^	1.34 (0.92-1.94)
Suicidal ideation	4881	0.81 (0.53-1.22)	2.11 (1.45-3.07)^f^	0.72 (0.47-1.11)	1.62 (1.09-2.42)^d^	1.37 (1.05-1.79)^d^	1.29 (0.95-1.75)
Suicide attempt^g^	6062	2.21 (1.05-4.65)^d^	2.77 (0.80-9.60)	1.63 (0.76-3.51)	1.42 (0.40-5.03)	1.81 (0.89-3.69)	1.34 (0.64-2.82)

Abbreviations: AOR, adjusted odds ratio; GAD, generalized anxiety disorder; MDD, major depressive disorder; PD, panic disorder; PTSD, posttraumatic stress disorder; SUD, substance use disorder.

^a^ Outcomes were measured at wave 1 of the STARRS (Study to Assess Risk and Resilience in Servicemembers) Longitudinal Study, approximately 5 years after participants completed the baseline New Soldier Study survey. The numbers vary because the models evaluate new onset of mental health problems and exclude soldiers who reported the specified outcome at baseline. Partial and full models also adjusted for age, sex, race and ethnicity, educational attainment, marital status, and site of basic combat training (all measured at baseline), as well as deployment history and military status at follow-up.

^b^ Indicates pre-enlistment history of internalizing disorder (yes if lifetime PTSD, MDD, mania/hypomania, GAD, or PD).

^c^ Indicates pre-enlistment history of externalizing disorder (yes if conduct disorder, oppositional defiant disorder, or SUD).

^d^ *P* < .05.

^e^ *P* < .01.

^f^ *P* < .001.

^g^ The full model of new onset of suicide attempt also included lifetime suicidal ideation at baseline as an independent variable, and that association was nonsignificant (AOR, 1.94; 95% CI, 0.82-4.59).

Subsequent models (full models in Table 2) added controls for baseline psychiatric comorbidity. Pre-enlistment impairing anger attacks were no longer associated with new onset of MDD and PD, with model results suggesting that those associations were explained by internalizing disorders at baseline. Pre-enlistment impairing anger attacks remained significantly associated with new onset of GAD (AOR, 1.75; 95% CI, 1.19-2.58; *P* < .001) and suicidal ideation (AOR, 1.62; 95% CI, 1.09-2.42; *P* = .02), although these associations were attenuated compared with those of the partial models. The association between pre-enlistment nonimpairing anger attacks and suicide attempt became nonsignificant when baseline psychiatric comorbidity was added to the model.

## Discussion

The present study examines the baseline correlates and psychiatric sequelae of anger attacks among new US Army soldiers. Compared with a previous study that found *DSM-IV* IED was highly prevalent in this sample (14.6%),^8^ we found that recurrent anger attacks that caused substantial life interference (resembling those that characterize *DSM-5* IED) were less common among new soldiers, with a prevalence of 5.75%. Impairing anger attacks were associated with markedly increased rates of lifetime psychiatric disorders, as well as an elevated risk of postenlistment new onset of MDD, GAD, PD, and suicidal ideation.

New soldiers with a history of impairing vs nonimpairing anger attacks reported more lifetime episodes, with approximately one-quarter reporting more than 50 anger attacks in their lifetimes. New soldiers with either type of anger attacks were more likely to be young, White, and male and less likely to be married or college-educated than those without a history of anger attacks. This adds to other evidence indicating that IED and related outcomes (eg, problematic anger, aggression) are associated with younger age, male sex, unmarried status, and lower educational/economic status.^23,24,25,26,27^ Results of previous research examining racial and ethnic differences in anger-related outcomes have been mixed, with some studies finding increased prevalence among individuals identifying as Black,^24^ Native American,^28^ and other race and ethnicity^2^ compared with White US individuals. These inconsistent findings suggest that more research is needed to clarify the association between race and ethnicity (and other sociodemographic characteristics) and anger-related outcomes, in both military and nonmilitary samples.

Compared with those without anger attacks, new soldiers with anger attacks were more likely to report lifetime mental disorders, suicidal ideation, and suicide attempt. Impairing anger attacks were associated with markedly elevated comorbidity rates (4-fold to 12-fold risk), whereas nonimpairing anger attacks were associated with a 2-fold to 4-fold risk of other mental health problems. This implies that recurrent anger attacks, which may be more readily apparent and subject to less stigma in this population compared with other emotional problems,^29,30^ signal higher likelihood of anxiety, mood, and stress-related disorders and SUDs in new soldiers. Moreover, when anger attacks cause substantial life interference, the odds of psychiatric comorbidities greatly increase, with particularly elevated (>10 times) risk of mania/hypomania, PD, GAD, and PTSD.

In prospective analyses adjusted for sociodemographic and service variables, new soldiers with impairing anger attacks were at increased risk of postenlistment onset of MDD, GAD, PD, and suicidal ideation. Soldiers who reported nonimpairing attacks did not exhibit higher risk of those mental health outcomes but had significantly elevated risk of a first suicide attempt after enlistment. (Although the risk also appeared elevated in those with impairing anger attacks, that association was nonsignificant, probably owing to smaller subgroup size.) These results imply that impairing anger attacks signal heightened vulnerability to developing anxiety disorders, depression, and suicidality after enlistment and that even anger attacks that are perceived as nonimpairing may indicate an increased risk of suicide attempt.

Further analyses investigated whether the associations between pre-enlistment anger attacks and later mental health problems were explained by co-occurring mental disorders at baseline. Notably, impairing anger attacks remained significantly associated with GAD and suicidal ideation, even after adjustment for pre-enlistment internalizing and externalizing disorders. Thus, detection of impairing anger attacks could aid in assessing risk of GAD and suicidality in soldiers, even when psychiatric history is known. This possibility is perhaps most significant given concerns about the Army suicide rate, which has risen in recent years.^31^

Future studies may help to shed light on potential mechanisms for the prospective associations observed in this study. It is unknown why impairing anger attacks would contribute uniquely to GAD and suicidal ideation, but not other emotional disorders. However, the occurrence of anger attacks seems to imply serious deficits in emotion regulation and social problem-solving. Theoretical accounts and empirical evidence implicate emotion regulation deficits in the etiology of GAD^32,33^ and social problem-solving deficits in the development of suicidal ideation^34^; thus, these may be fruitful avenues for further inquiry. Interventions to reduce anger attacks (or emotional regulation/problem-solving deficits) might help to prevent GAD and suicidal ideation in soldiers.

The study findings appear to support the addition of the criterion for IED diagnosis in *DSM-5* requiring that anger attacks cause marked distress, impairment in occupational or interpersonal functioning, or financial or legal consequences.^9^ Such a requirement indeed appears to identify more severe psychopathology, as indicated by more pervasive symptoms (eg, more lifetime anger attacks), markedly higher psychiatric comorbidity, and elevated incidence of subsequent anxiety disorders, mood disorders, and suicidal ideation in those with impairing anger attacks. However, our data also indicate that the issue of insight into interference caused by anger attacks is worth considering, because a sizable proportion of soldiers who reported minimal life interference reported high frequency of anger attacks (eg, 11.6% reported >50 lifetime attacks). Specific questions about interpersonal and work-related problems may be helpful in characterizing impairment related to anger attacks.

### Limitations

These results should be considered in light of several limitations. Mental health variables, including history of anger attacks, were assessed using self-report measures, which are vulnerable to inaccurate recall and response bias. Suicide attempts were rarely reported, and our study may have been underpowered to detect significant associations between baseline variables and this outcome. In addition, although we included a number of potential confounds as covariates, it is possible that other, unmeasured variables explain the associations between history of anger attacks at baseline and mental health status at follow-up. Finally, this sample is representative of new Army soldiers; these results may not generalize to more experienced soldiers, members of other service branches, or the general adult population.

## Conclusions

As shown in this cohort study, a history of anger attacks is common in new soldiers and associated both cross-sectionally and prospectively with a range of mental health problems. Pre-enlistment anger attacks that lead to substantial life interference, such as those characterizing *DSM-5* IED, may be a risk marker for GAD, MDD, PD, and suicidality after enlistment. Moreover, detection of impairing anger attacks could aid in psychiatric risk assessment in new soldiers, even when pre-enlistment psychiatric history is known. Further investigation of anger-related problems in the military population is imperative and may inform future mental health monitoring and intervention.

## Notes

Supplement. **eMethods.** Additional Information About STARRS-Longitudinal Study (STARRS-LS) Sampling
