# Supplementary material for: Pre-enlistment Anger Attacks and Postenlistment Mental Disorders and Suicidality Among US Army Soldiers
Source: JAMA Netw Open. 2021 Sep 27;4(9):e2126626. doi: 10.1001/jamanetworkopen.2021.26626 (PMC8477262; doi:10.1001/jamanetworkopen.2021.26626)
Supplement: Supplement. — eMethods. Additional Information About STARRS-Longitudinal Study (STARRS-LS) Sampling [file jamanetwopen-e2126626-s001.pdf]

Supplemental Online Content

Smith DM, Meruelo A, Campbell-Sills L, et al; Army STARRS Team. Pre-enlistment anger attacks and postenlistment mental disorders and suicidality among US Army soldiers. *JAMA Netw Open*. 2021;4(9):e2126626. doi:10.1001/jamanetworkopen.2021.26626

eMethods. Additional Information About STARRS–Longitudinal Study (STARRS-LS) Sampling

This supplementary material has been provided by the authors to give readers additional information about their work.

© 2021 Smith DM et al. *JAMA Network Open.*

eMethods. Additional Information About STARRS–Longitudinal Study (STARRS-LS) Sampling

Soldiers who were eligible for inclusion in STARRS-LS included 72,387 soldiers who participated in one or more Army STARRS surveys and consented to linkage of their survey data to their Army/Department of Defense administrative records. The aims of STARRS-LS involved a focus on special operation forces (SOF), Guard/Reserve members, and female soldiers; therefore, the eligible baseline sample was divided into three strata. Stratum 1 was comprised of 22,207 Army STARRS survey respondents who reported lifetime mental disorders or suicidal thoughts/behavior. Stratum 2 was comprised of 26,823 additional respondents (i.e., with no reported history of mental health problems) who were SOF, Guard/Reserve members, or female. Stratum 3 included the remaining 23,357 respondents (i.e., male, Regular Army, not SOF). Due to limited follow-up capacity, the final STARRS-LS Wave 1 sample (N=51,963) was based on: (a) 100% of respondents from Stratum 1, (b) 100% of SOF soldiers, (c) a probability sample of 67% from the rest of Stratum 2, and (d) a probability sample of 50% from Stratum 3. This resulted in 22,846 STARRS-LS Wave 1 respondents from the first stratum; 26,184 respondents from the second stratum; and 23,357 respondents from the third stratum. STARRS-LS Wave 1 data are weighted to adjust for the differential sampling. The prospective portion of the current analysis included 6,216 soldiers from the New Soldier Study sample (as described in Rosellini et al., 2015), whose New Soldier Study data were successfully linked to STARRS-LS wave 1 data.

© 2021 Smith DM et al. *JAMA Network Open.*
